# Application of amplicon-based targeted sequencing with the molecular barcoding system to detect uncommon minor *EGFR* mutations in patients with treatment-naïve lung adenocarcinoma

**DOI:** 10.1186/s12885-019-5374-1

**Published:** 2019-02-26

**Authors:** Kei Namba, Shuta Tomida, Takehiro Matsubara, Yuta Takahashi, Eisuke Kurihara, Yusuke Ogoshi, Takahiro Yoshioka, Tatsuaki Takeda, Hidejiro Torigoe, Hiroki Sato, Kazuhiko Shien, Hiromasa Yamamoto, Junichi Soh, Kazunori Tsukuda, Shinichi Toyooka

**Affiliations:** 1 Departments of Thoracic, Breast and Endocrinological Surgery, Okayama University Graduate School of Medicine, Dentistry and Pharmaceutical Sciences, 2-5-1 Shikata-cho, Kita-ku, Okayama City, 700-8558 Japan; 2 Department of Biobank, Okayama University Graduate School of Medicine, Dentistry and Pharmaceutical Sciences, 2-5-1 Shikata-cho, Kita-ku, Okayama City, 700-8558 Japan; 3 Okayama University Hospital Biobank, Okayama University Hospital, 2-5-1 Shikata-cho, Kita-ku, Okayama City, 700-8558 Japan; 4 Department of Gastroenterological Surgery, Okayama University Graduate School of Medicine, Dentistry and Pharmaceutical Sciences, 2-5-1 Shikata-cho, Kita-ku, Okayama City, 700-8558 Japan; 5 Department of Clinical Pharmacy, Okayama University Graduate School of Medicine, Dentistry and Pharmaceutical Sciences, 2-5-1 Shikata-cho, Kita-ku, Okayama City, Okayama 700-8558 Japan

**Keywords:** Non-small cell lung cancer, Epidermal growth factor receptor, Compound mutations, Molecular barcoding, Genomic heterogeneity, Patient genotype, Next-generation sequencing, Molecular barcoding, *EGFR*, Non-small cell lung cancer, Treatment-naïve, Sequence artifact, Clinical sequencing, Uncommon mutation, Mutation detection

## Abstract

**Background:**

In lung cancer, epidermal growth factor receptor (EGFR) tyrosine kinase inhibitor sensitizing mutations co-existing with rare minor *EGFR* mutations are known as compound mutations. These minor *EGFR* mutations can lead to acquired resistance after *EGFR* tyrosine kinase inhibitor treatment, so determining the mutation status of patients is important. However, using amplicon-based targeted deep sequencing based on next-generation sequencing to characterize mutations is prone to sequencing error. We therefore assessed the benefit of incorporating molecular barcoding with high-throughput sequencing to investigate genomic heterogeneity in treatment-naïve patients who have undergone resection of their non-small cell lung cancer (NSCLC) *EGFR* mutations.

**Methods:**

We performed amplicon-based targeted sequencing with the molecular barcoding system (MBS) to detect major common *EGFR* mutations and uncommon minor mutations at a 0.5% allele frequency in fresh–frozen lung cancer samples.

**Results:**

Profiles of the common mutations of *EGFR* identified by MBS corresponded with the results of clinical testing in 63 (98.4%) out of 64 cases. Uncommon mutations of *EGFR* were detected in seven cases (10.9%). Among the three types of major *EGFR* mutations, patients with the G719X mutation had a significantly higher incidence of compound mutations than those with the L858R mutation or exon 19 deletion (*p* = 0.0052). This was validated in an independent cohort from the Cancer Genome Atlas dataset (*p* = 0.018).

**Conclusions:**

Our findings demonstrate the feasibility of using the MBS to establish an accurate NSCLC patient genotype. This work will help understand the molecular basis of *EGFR* compound mutations in NSCLC, and could aid the development of new treatment modalities.

**Electronic supplementary material:**

The online version of this article (10.1186/s12885-019-5374-1) contains supplementary material, which is available to authorized users.

## Background

Lung cancer is the leading cause of cancer mortality worldwide, and non-small cell lung cancer (NSCLC) accounts for more than 85% of all lung cancers [1, 2]. The molecular profiles of NSCLC have been extensively analyzed, and revealed that activating mutations in the epidermal growth factor receptor gene (*EGFR*) are found in approximately 10–15% of Caucasian patients and 30–40% of Asian patients with lung adenocarcinoma [3–8]. The successful development of molecular targeting agents that inhibit growth signals from driver mutations has improved the treatment outcome of adenocarcinoma patients with *EGFR* mutations [9–11].

Recently, several reports have demonstrated the existence of compound *EGFR* mutations, where an EGFR tyrosine kinase inhibitor (EGFR-TKI) sensitizing mutation coexists with uncommon mutations such as T790 M, E709G/K, R776H, and L844 V [12–15]. These rare minor mutations cause acquired resistance after EGFR-TKI treatment [14, 15], leading to disease progression. Thus, it is important to understand the status of uncommon as well as common mutations.

The clinical implementation of amplicon-based targeted deep sequencing based on next-generation sequencing (NGS) technologies has been widely adopted [16], and enables the analysis of small amounts of input DNA such as those extracted from formalin-fixed paraffin-embedded samples. However, a major problem of high-throughput DNA sequencing is the increased rate of errors introduced during sample preparation and sequencing, resulting in difficulties in determining the true genotype status, especially for infrequent mutant allele [17, 18]. The molecular barcoding system (MBS) aims to resolve the impact of enrichment and sequencing artifacts, and has the potential to improve the mutation detection accuracy [18–20], enabling us to understand genomic heterogeneity in NSCLC.

In the current study, we used a high-sensitivity, amplicon-based targeted deep sequencing method that incorporates the MBS to investigate genomic heterogeneity in treatment-naïve NSCLC patients with *EGFR* mutations who have undergone resection of their cancers.

## Methods

### Tumor preparation and DNA extraction

We retrospectively analyzed 590 consecutive patients who underwent surgical resection for primary lung cancer at Okayama University Hospital between January 2012 and December 2015. The inclusion criteria were as follows: (1) treatment-naïve before surgery, (2) histologic documentation of adenocarcinoma, and (3) a positive *EGFR* mutation status tested using a standard conventional method (peptide nucleic acid–locked nucleic acid PCR clamp method, or clinical testing) [21]. In the PCR clamp method, it is possible to detect the following *EGFR* mutations; Exon 19 deletions, L858R, L861Q, T790 M and G719A/S/C. Patients without surgical pathology reports or stored fresh–frozen samples were excluded.

Out of the remaining 531 treatment-naïve patients, 415 (78%) were diagnosed with adenocarcinoma (206 men and 209 women). Based on clinical tests, 169 patients (59 [35%] men and 110 women) were shown to be *EGFR* mutation-positive (Additional file 1: Figure S1). Sixty-four of these 169 *EGFR* mutation-positive adenocarcinomas were randomly selected and sequenced with the MBS.

Frozen lung cancer samples were procured at the time of surgery, immediately frozen in liquid nitrogen, and stored until use. Genomic DNA from available fresh-frozen tissue samples was extracted using the phenol-chloroform method. The DNA quality and concentrations were assessed using the Qubit 3.0 Fluorometer (Thermo Fisher Scientific, Waltham, MA, USA).

### Library preparation using the MBS

Target enrichment was performed on 100 ng of input DNA using the HaloPlex^HS^ system (Agilent Technologies, Santa Clara, CA), which is a high-sensitivity, amplicon-based targeted sequencing method incorporating the MBS in the DNA library. Library was prepared based on the manufacturer’s protocol for ClearSeq Cancer HS, ILM, which was designed to identify somatic variants in 47 cancer-related genes (Additional file 2: Table S1) targeting known COSMIC hotspots found to be associated with a broad range of cancer types as well as published drug targets (https://www.agilent.com/en/product/amplicon-based-next-generation-sequencing-(ngs)/amplicon-target-capture-kits/haloplexhs-custom-kits-232856). Sequencing was performed using the MiSeq (v3 600-cycle kit (Illumina, San Diego, CA, USA) according to the manufacturer’s recommendations for paired-end sequencing (2 × 300 cycles) and the HiSeq 2500 Rapid mode 300-cycle (Illumina). Patient data were collected retrospectively from clinical records.

**Fig. 1:**
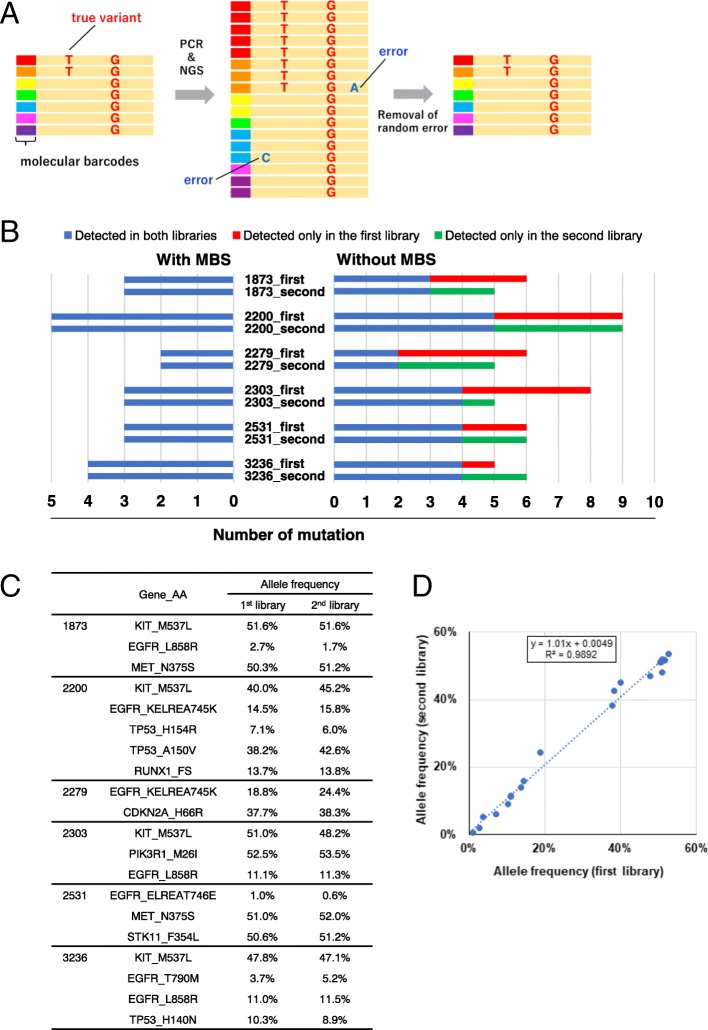
Reproducibility of MBS. **a** Outline of the molecular barcoding system. **b** Technical replication of 6 samples from the evaluation cohort. Comparison of the reproducibility of targeted deep sequencing with or without the molecular barcoding system is displayed. **c** Detected allele frequency of variables with molecular barcoding between technical replications. **d** The approximately liner relationship between technical replications

The generated sequences were processed in-house using Agilent SureCall software, which is used exclusively with the HaloPlex^HS^ system. Briefly, the molecular barcode analysis consisted of the following steps: first, the individual DNA molecules were tagged with molecular barcodes during library preparation. Next reads were aligned to the same genomic coordinates. Then, the reads with an identical molecular barcode were grouped into each molecular family. Finally, the base sequences for a molecular barcode were consolidated to one read per molecular barcode (random error, including PCR errors and sequence errors, were removed) (Fig. 1a).

### Direct sequencing

Rare *EGFR* mutations were confirmed using standard DNA sequencing techniques with the direct sequencing of several *EGFR* exons. Briefly, DNA was isolated from the sample, quantified, and subjected to PCR using primers for exons 15 to 21 of *EGFR* (Additional file 3: Table S2). The PCR products were then analyzed using bidirectional direct DNA sequencing.

### Analysis of The Cancer Genome Atlas (TCGA) datasets

TCGA datasets were analyzed and downloaded via the cBioPortal (http://www.cbioportal.org). RNA-Seq gene expression data for TCGA samples were collected from the Genomic Data Commons Data Portal (https://portal.gdc.cancer.gov). Microarray gene expression data for 127 lung adenocarcinomas with *EGFR* mutations from the Japanese National Cancer Center Research Institute were collected from Gene Expression Omnibus (https://www.ncbi.nlm.nih.gov/geo/) as GSE31210. Functional analyses of the TCGA samples were performed using gene set enrichment analysis (GSEA) (Molecular Signatures Database v5.0) [22].

### Statistical analysis

Statistical analyses were performed using GraphPad Prism 7 (GraphPad Software). Overall survival (OS) rates and recurrence-free survival (RFS) rates were calculated using the Kaplan–Meier method, and differences in survival rates between the groups were compared using the log-rank test. A value of *p* < 0.05 was considered statistically significant.

## Results

### Reproducibility of MBSs

To confirm the reproducibility of targeted deep sequencing with or without MBSs, we examined the technical reproducibility using six samples (Additional file 4: Table S3). The first and the second libraries were prepared independently from the same DNA extraction of each samples. Using MBS, the exact same results were obtained for the first and the second libraries of #1873, such as three mutations for *KIT*, *EGFR* and *MET* (Fig. 1b, c). Without MBS, however, three orphan mutations were detected additionally for the first library as well as two orphan mutations for the second library of #1873, resulting in the detection of six matched mutations plus five orphan mutations, corresponding to a reproducibility of 55% (6/11) (Additional file 5: Table S4). In the same way, 100% reproducibility was obtained for all the six samples by using MBS, while on average 58% reproducibility was observed without MBS, ranging from 36% (4/11) in patient #2279 to 73% (8/11) in patient #3236 (Fig. 1b, Additional file 5: Table S4). Also, the allele frequencies of *EGFR* mutations detected with MBS were comparable between the two libraries across a wide range from around 1 to 50%, including 0.6% of EGFR mutation for the second library of #2351 (Fig. 1c), with significant correlation between the two libraries (R^2^ = 0.989, Fig. 1d), and thus demonstrating the reproducibility of MBS in quantifying allele frequency of *EGFR* mutations in lung cancer.

### Reducing potential sequence artifact using MBS

**Fig. 2:**
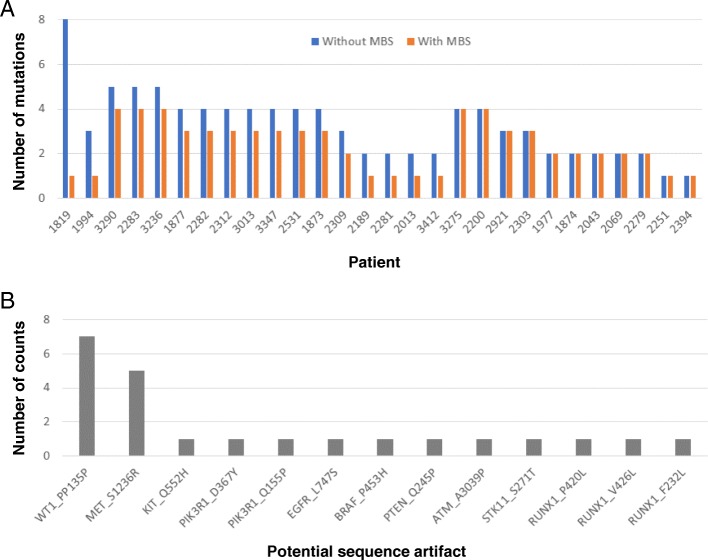
Error suppression of MBS. **a** Detected genetic alterations with/without the use of molecular barcoding system (*n* = 28). **b** Potential sequence artifact found in samples sequenced without MBS

Next, to evaluate the ability of MBS to reduce potential sequence artifacts, we performed targeted sequencing with or without MBS by expanding the samples to 28 (Additional file 4: Table S3). In order not to overestimate the ability of MBS to reduce potential sequence artifacts, systematic sequence artifacts, which were found in more than half of the sample, were filtered out before we compared the results. On average, 2.4 genetic alterations were detected with MBS (ranging from 1 to 4), while 3.3 genetic alterations were detected without MBS (ranging from 1 to 8) (Fig. 2a). One or more potential sequence artifacts (ranging from 1 to 7) were reduced in 17 of the 28 samples (61%) with MBS. In addition, 11 out of 13 potential sequence artifacts were singletons, which were found in only one sample resulting in difficulty to distinguish those artifacts from the actual genetic alterations (Fig. 2b). Together, these results support the ability of MBS to reduce potential sequence artifacts.

### Determination of genomic heterogeneity

**Fig. 3:**
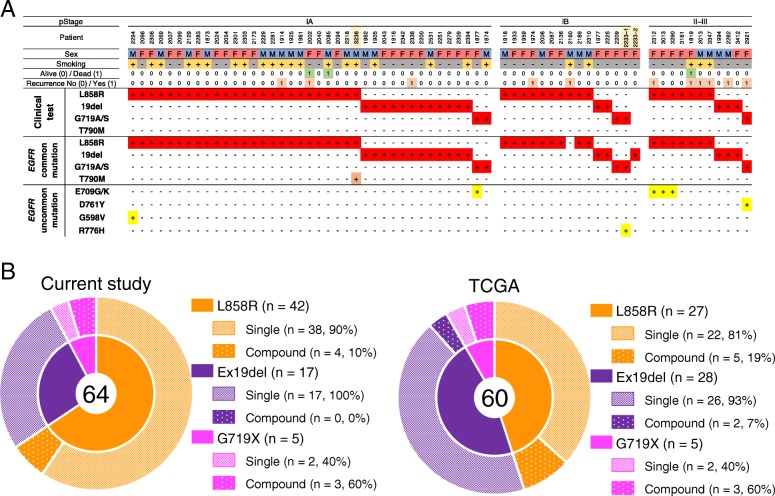
Comparison of *EGFR* mutations detected using clinical sequencing and targeted deep sequencing. **a** Mutation summary of clinical sequencing cohort (*n* = 64). **b** Incidence of concomitant *EGFR* uncommon mutations in patient with L858R (orange), exon 19 deletion (purple), and G719X (pink). Patients with single mutation (pale shaded) and compound mutation (solid with dot) were outlined

To evaluate the concordance between *EGFR* mutations detected using clinical tests and targeted deep sequencing with MBS, DNA from 64 patients with adenocarcinoma was sequenced. The clinicopathological characteristics of the patients are summarized in Additional file 4: Table S3. All patients had histological confirmation of adenocarcinoma, and were classified as pathological stage IA (*n* = 39, 61%), IB (*n* = 14, 22%), and II–III (*n* = 11, 17%) according to the TNM Classification of Malignant Tumors seventh edition. Examination of the status of common *EGFR* mutations identified via clinical tests revealed 42 (66%) L858R mutations, 17 (27%) exon 19 deletions, and five (8%) G719S/A mutations. Targeted deep sequencing using MBS detected common *EGFR* mutations in 63 of the 64 patients (98.4%) (Fig. 3a, Additional file 4: Table S3). Using MBS, we detected a T790 M mutation with an allele frequency of 3.7% and an L858R mutation with an allele frequency of 11.0% in patient #3236. In this patient, the T790 M mutation was not detected by routine clinical tests despite its inclusion in the examination. Instead, we confirmed the existence of T790 M in this patient using direct sequencing. From patient #2233, we simultaneously acquired two samples (#2233–1 and #2233–2) from different lobes of the lung. The *EGFR* mutations detected from these tumors were G719S and R776H mutations from one lesion (#2233–1) and an exon 19 deletion from another (#2233–2), indicating different tumor origins.

### Uncommon *EGFR* mutations

Of 64 patients, uncommon *EGFR* mutations were detected in seven cases (10.9%) (Fig. 3a, Table 1). The variant allele frequencies of uncommon *EGFR* mutations were 6.8–66%. Four of the seven cases possessed an exon 18 E709G mutation together with an L858R mutation (n = 3) or a G719S mutation (*n* = 1). The other cases had an exon 20 R776H mutation with a G719S mutation (n = 1), an exon 19 D761Y mutation with a G719S mutation (n = 1), and an exon 15 G598 V mutation, a known pathogenic mutation localized to the extracellular domain [23], with an L858R mutation (n = 1). These uncommon mutations were confirmed by direct sequencing (Additional file 6: Figure S2). The frequency of common mutation and uncommon mutation was almost identical in five cases which possessed E709G mutation or G598 V mutation. On the other hand, in the case of G719S/R776H and G719S/D761Y, the frequency of common mutation and uncommon mutation differed by more than 15% (31% vs 48, 19% vs 66%). We analyzed whether the differences of frequency differ by mutation, such as L858R vs. G719S, but, due to the small number of cases, statistically significant differences were not observed.

**Table 1:** Allele frequency of common and uncommon EGFR mutations

Patient	Common mutation	Frequency (%)	Uncommon mutation	Frequency (%)	Difference (%)
2294	L858R	6.56	G598 V	6.8	0.24
2312	L858R	13.3	E709G	13.9	0.6
3013	L858R	20.4	E709G	20.4	0
3290	L858R	20.2	E709G	18.4	1.8
1877	G719S	11.8	E709G	12.3	0.5
2233	G719S	30.9	R776H	48.1	17.2
2921	G719A	19.4	D761Y	65.9	46.5

We further analyzed the potential correlations among the clinicopathological characteristics of patients with *EGFR* mutations. There were no differences in age, gender, or smoking habit distribution between the patients with a single mutation and those with a compound mutation, however, among patients with the three types of *EGFR* common mutations, those with G719X (60%, 3/5) had a significantly higher incidence of concomitant uncommon *EGFR* mutations than those with L858R (9.5%, 4/42) or exon 19 deletions (0%, 0/17) (*p* = 0.0052) (Table 2, Additional file 7: Table S5), as previously reported [24–26]. To validate these findings with an independent cohort, we analyzed the TCGA Pan-Lung cancer dataset, including 660 lung adenocarcinomas (LUAD) [8]. In 104 LUAD patients, 123 *EGFR* mutations were detected. G719X, L858R, and exon 19 deletion mutations were found in five, 27, and 28 LUAD patients, respectively. Notably, patients with the G719X mutation had a significantly higher incidence of compound mutations (60%, 3/5) compared with those with either L858R (19%, 5/27) or the exon 19 deletion mutation (7.1%, 2/28) (*p* = 0.018; Fig. 3b). These results strongly support our findings that patients with the G719X mutation are likely to have *EGFR* compound mutations.

**Table 2:** Clinical and pathologic characteristics of the cases with EGFR single/compound mutation

Parameter	Subtype	Single (*n* = 57)	Compound (*n* = 7)	*P*-value
^a^Mean age (mean ± SD); yrs		66.4 ± 9.6	66.7 ± 9.3	0.94
^b^Sex, n (%)	Male	23	1	0.24
	Female	34	6	
^b^Smoking habit, n (%)	No	31	6	0.22
	Yes	26	1	
^b^EGFR mutation, n (%)	L858R	38	4	0.0052*
	Exon19del	17	0	
	G719A/S	2	3	
^a^Tumor size (mean ± SD); mm		22.5 ± 8.9	33.7 ± 23.3	0.014*
^b^pStage, n (%)	IA	37	2	0.012*
	IB	13	1	
	IIA-IIIB	7	4	

^a^*P*-value was obtained from t-test

^b^*P*-value was obtained from Fisher’s test

A value of *p* < 0.05 was marked "*"

To shed light on the molecular mechanisms, we compared gene expression profiles between patients with compound *EGFR* mutations and those with single *EGFR* mutations for respective mutations. A total of 44 RNA-Seq gene expression profiles were available from the TCGA LUAD dataset (L858R; *n* = 21, exon 19 deletions; *n* = 20, G719X; *n* = 3). A total of 11 genes were significantly altered in patients with the G719X compound mutation, while only three were significantly repressed in patients with the L858R compound mutation, and six genes were altered in the patient with the exon 19 deletion compound mutation (Additional file 8: Figure S3A, B). In addition, we performed GSEA using hallmark gene sets, revealing that the following gene sets were positively correlated with the patient with the G719X compound mutation, such as “TGF beta signaling” and “UV response DN”, while the following gene sets were negatively correlated, such as “reactive oxygen species pathway” and “pancreas beta cells” (Additional file 8: Figure S3C).

## Discussion

In the current study, we demonstrated the feasibility of a molecular barcoding system to reduce potential sequence artifacts, especially during targeted deep sequencing. The correspondence rate between the common *EGFR* mutation profiles produced by MBS and clinical tests was 98.4% (63 out of 64 cases). The discordance was observed in only one patient #2160, in whom L858R mutation was positive at clinical testing but negative at NGS. In clinical testing, gDNA was extracted from FFPE sample, whereas gDNA from fresh frozen sample was used for NGS. These differences of samples from the same patient, in other words tumor heterogeneity may be the cause of the discordance.

We identified a de novo T790 M mutation that could not be detected by clinical tests, representing a potential advantage of this system. We also used molecular barcoding to investigate the status of uncommon *EGFR* mutations coexisting with common mutations in treatment-naïve primary lung cancer patients. In the past decade, different frequencies of uncommon *EGFR* mutations found together with common mutations have been documented, ranging from 3.6 to 14.8% [14, 27–30]. Moreover, several NGS-based studies have reported an incidence of *EGFR* compound mutations of 3.0 to 16% in *EGFR*-mutated lung cancers [25, 31]. In our study, uncommon *EGFR* mutations were detected in 10.9% (7/64) of cases.

We also showed that *EGFR* G719X-harboring cases had a significantly higher incidence of concomitant uncommon *EGFR* mutations than those with L858R or exon 19 deletion mutations. This result supported the findings of large-scale analyses of over 1000 cases reported by Illei et al. [25] and Wu et al. [30], suggesting that patients with the G719X mutation are likely to have *EGFR* compound mutations. In addition, Kohsaka et al. has reported that more than 90% of the G719 mutations existed as compound mutations while only 19.5 and 4.7% of L858R mutations and exon 19 deletions respectively harbor compound mutations [24].

Lung cancer patients harboring G719X mutations were reported to have lower sensitivities to first-generation EGFR-TKIs and shorter survival times than those with L858R mutations or an exon 19 deletion [32, 33]. Previously, we described the first known case of an individual with a combination of D761Y and L858R mutations who did not respond to gefitinib, which was confirmed by other groups [34, 35]. Some germline *EGFR* mutations, including T790 M, V843I, and R776H, have been considered to predispose subjects to familial lung cancer and exhibit clinical resistance to EGFR-TKI [36–39]. Altogether, it is clinically important to know the mutation profile of both uncommon and common *EGFR* mutations for patients with NSCLC.

## Conclusion

Amplicon sequencing incorporating an MBS is a feasible approach to determine the mutation profile of both uncommon and common *EGFR* mutations. Uncovering the true genotype using an accurate NGS platform will enable the development of proper therapeutic strategies for NSCLC.

## Additional files

Additional file 1: **Figure S1.** Patients population. Of 531 patients without any treatment before surgery, 415 (78%) were diagnosed as having adenocarcinoma. Based on clinical tests, 169 patients were diagnosed as *EGFR*-mutation positive. Of the 169 *EGFR*-mutation positive adenocarcinomas, 64 adenocarcinoma specimens were randomly selected and sequenced with MBS. (PDF 315 kb)

Additional file 2: **Table S1.** ClearSeq Cancer Panel Gene List. (XLSX 10 kb)

Additional file 3: **Table S2.** DNA sequencing primer. (XLSX 10 kb)

Additional file 4: **Table S3.** Patient characteristics. (XLSX 14 kb)

Additional file 5: **Table S4.** Reproducibility of targeted deep sequencing without MBS. (XLSX 12 kb)

Additional file 6: **Figure S2.** *EGFR* uncommon mutations detected by targeted sequencing. All the uncommon *EGFR* mutations detected in 7 cases were confirmed by direct sequencing. ECD, Extra-cellular Domain. (PDF 1223 kb)

Additional file 7: **Table S5.** Patient characteristics. (XLSX 10 kb)

Additional file 8: **Figure S3.** Molecular profiles of *EGFR* compound mutations. (A) In patients with the G719X compound mutation, five genes were highly induced while six genes were significantly repressed. In total, 11 genes were significantly altered in patient with the G719X compound mutation, while only 3 genes were significantly repressed in patient with the L858R compound mutation and 6 genes were altered in patient with the Ex19del compound mutation. (B) All the five significantly induced genes were unique for patient with the G719X compound mutation, while three out of six significantly down-regulated genes were overlapped with those of patient with the Ex19del compound mutation. (C)We performed GSEA using hallmark gene sets, revealing that several gene sets were correlated with the patient with the G719X compound mutation. (PDF 168 kb)

## Abbreviations

COSMIC: Catalogue of somatic mutations in cancer
EGFR: Epidermal growth factor receptor
MBS: Molecular barcoding system
NGS: Next-generation sequencing
NSCLC: Non-small cell lung cancer
PCR: Polymerase chain reaction
TCGA: The Cancer Genome Atlas
TKI: Tyrosine kinase inhibitor
