# Supplementary figures and images for: Application of amplicon-based targeted sequencing with the molecular barcoding system to detect uncommon minor EGFR mutations in patients with treatment-naïve lung adenocarcinoma
Source: BMC Cancer. 2019 Feb 26;19:175. doi: 10.1186/s12885-019-5374-1 (PMC6390598; doi:10.1186/s12885-019-5374-1)

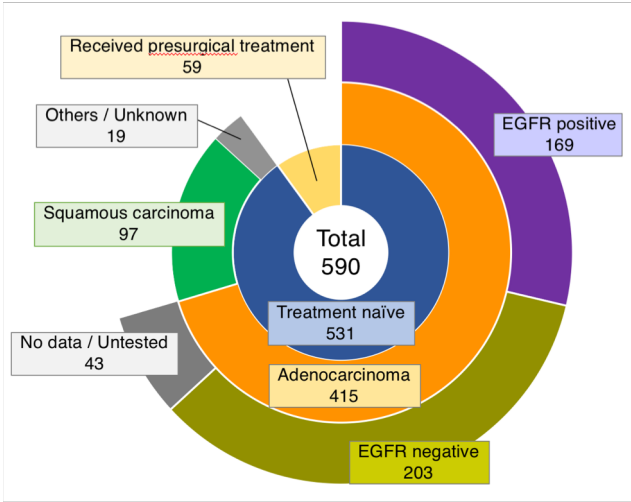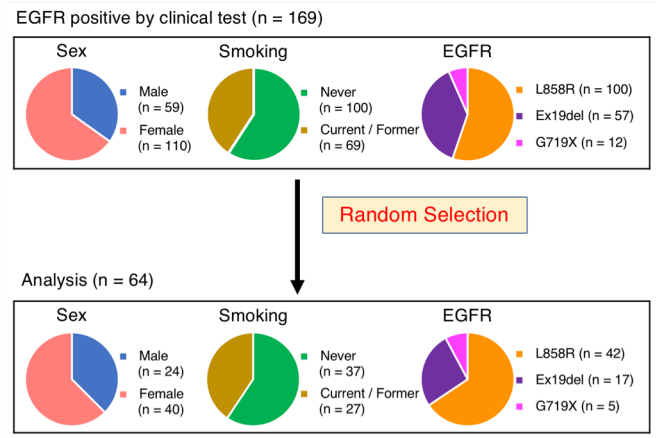

Namba et al. Fig. S1

Supplement: Supplementary file 1 — Figure S1. Patients population. Of 531 patients without any treatment before surgery, 415 (78%) were diagnosed as having adenocarcinoma. Based on clinical tests, 169 patients were diagnosed as EGFR-mutation positive. Of the 169 EGFR-mutation positive adenocarcinomas, 64 adenocarcinoma specimens were randomly selected and sequenced with MBS. (PDF 315 kb) [file 12885_2019_5374_MOESM1_ESM.pdf]

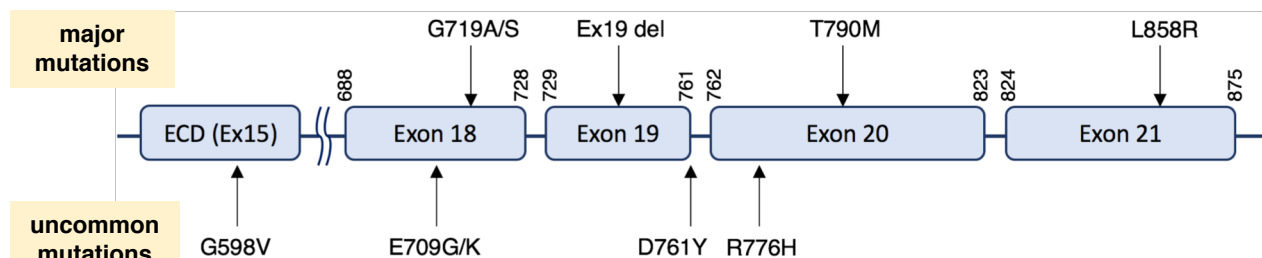

1877

E709K (c.2125G>A)

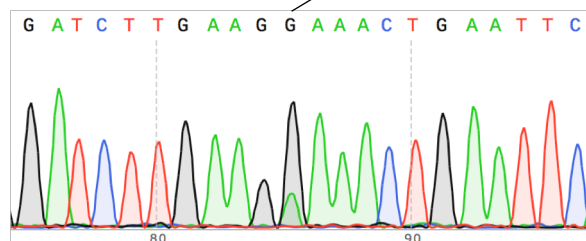

2312

E709G (c.2126A>G)

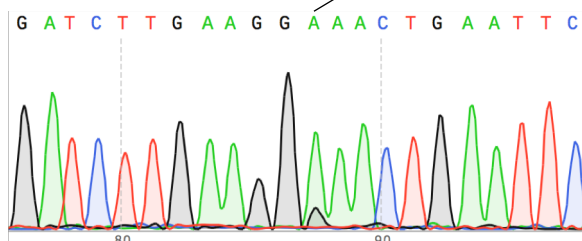

3013

E709G (c.2126A>G)

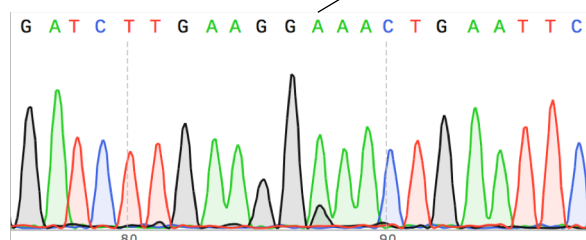

3290

E709G (c.2126A>G)

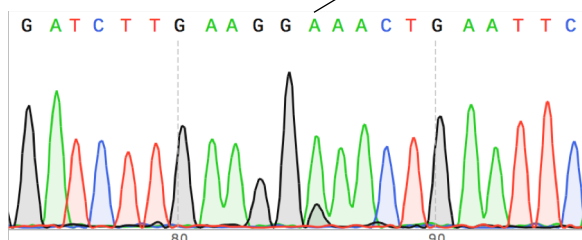

2921

D761Y (c.2281G>T)

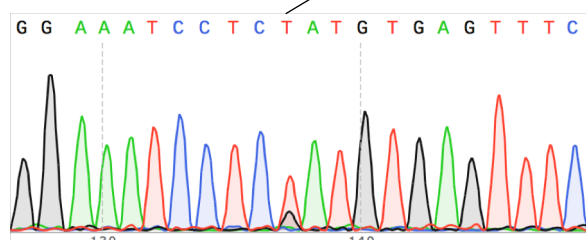

2233-1

R776H (c.2327G>A)

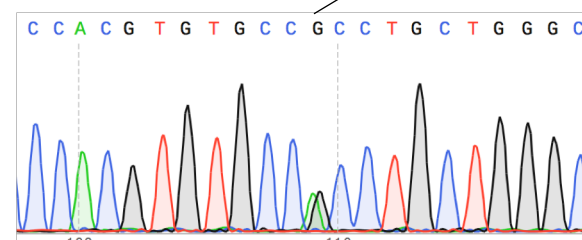

2294

G598V (c.1793G>T)

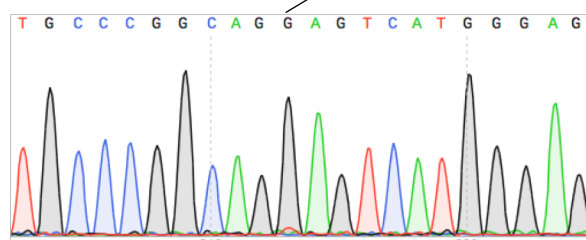

3236

T790M (c.2369C>T)

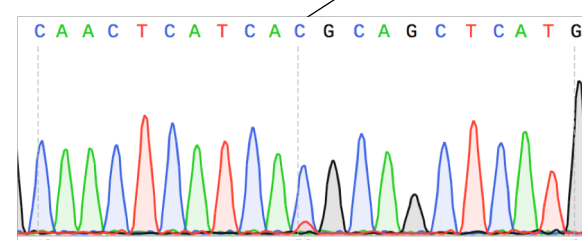

Supplement: Supplementary file 6 — Figure S2. EGFR uncommon mutations detected by targeted sequencing. All the uncommon EGFR mutations detected in 7 cases were confirmed by direct sequencing. ECD, Extra-cellular Domain. (PDF 1223 kb) [file 12885_2019_5374_MOESM6_ESM.pdf]
