# Supplementary material for: Application of amplicon-based targeted sequencing with the molecular barcoding system to detect uncommon minor EGFR mutations in patients with treatment-naïve lung adenocarcinoma
Source: BMC Cancer. 2019 Feb 26;19:175. doi: 10.1186/s12885-019-5374-1 (PMC6390598; doi:10.1186/s12885-019-5374-1)
Supplement: Supplementary file 8 — Figure S3. Molecular profiles of EGFR compound mutations. (A) In patients with the G719X compound mutation, five genes were highly induced while six genes were significantly repressed. In total, 11 genes were significantly altered in patient with the G719X compound mutation, while only 3 genes were significantly repressed in patient with the L858R compound mutation and 6 genes were altered in patient with the Ex19del compound mutation. (B) All the five significantly induced genes were unique for patient with the G719X compound mutation, while three out of six significantly down-regulated genes were overlapped with those of patient with the Ex19del compound mutation. (C)We performed GSEA using hallmark gene sets, revealing that several gene sets were correlated with the patient with the G719X compound mutation. (PDF 168 kb) [file 12885_2019_5374_MOESM8_ESM.pdf]

A

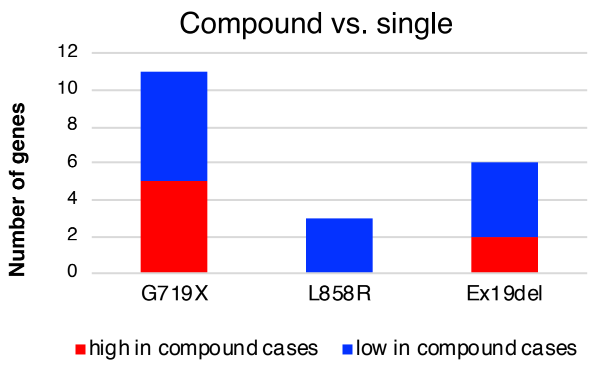

B

Compound vs. single

	G719X	L858R	Ex19del
HMGA2	6.4	ND	ND
MIR205HG	5.6	ND	ND
LY6D	4.4	ND	ND
PADI1	1.8	ND	ND
NXPH4	1.3	ND	ND
GSTM1	ND	ND	2.2
IGKV6-21	-1.3	ND	-1.5
IGHV4-4	-2.4	-2.1	1.0
CDKN2A	-2.5	ND	ND
FOXD1	-3.5	ND	ND
FGB	-6.3	ND	-7.2
SST	-6.5	ND	-6.1
PSPHP1	ND	-2.7	ND
CALML5	ND	-10.9	-7.3

C

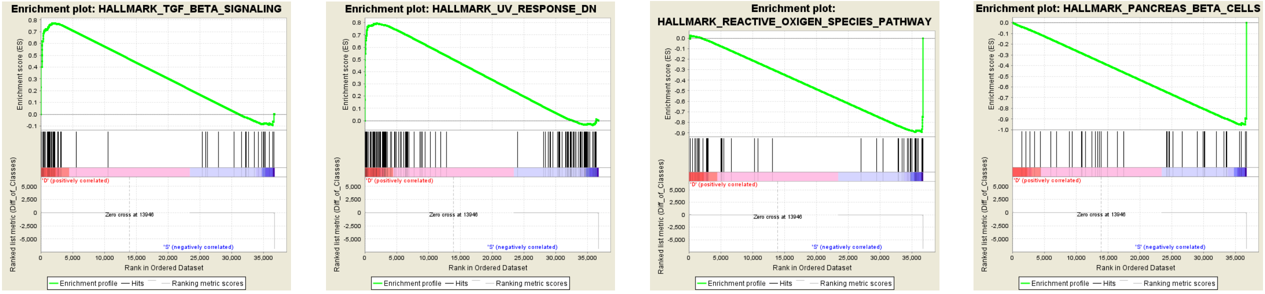

TGF-beta-signaling UV-response Reactive-oxigen Pancreas_beta_cell

Namba et al. Fig. S3
